# Greening healthcare and slashing carbon emissions through telemedicine: a cross-sectional study from over 50 thousand remote consults at a leading tertiary hospital

**DOI:** 10.3389/fdgth.2025.1497770

**Published:** 2025-05-30

**Authors:** Roberto Nunes Umpierre, Rita Mattiello, Carlos André Aita Schmitz, Enrique Falceto de Barros, Rodolfo Souza da Silva, Marcelo Rodrigues Gonçalves, José Roberto Goldim

**Affiliations:** ^1^ TelessaúdeRS-UFRGS, Universidade Federal do Rio Grande do Sul (UFRGS), Porto Alegre, Brazil; ^2^ Faculdade de Medicina, UFRGS, Porto Alegre, Brazil; ^3^ Programa de Pós-graduação em Ciências Médicas da UFRGS, Porto Alegre, Brazil; ^4^ Hospital de Clínicas de Porto Alegre, Porto Alegre, Brazil; ^5^ Programa de Pós-graduação em Epidemiologia da UFRGS, Porto Alegre, Brazil; ^6^ Universidade de Caxias do Sul (UCS), Caxias do Sul, Brazil; ^7^ WONCA Working Party on Planetary Health, Brussels, Belgium; ^8^ Laboratório de Pesquisa em Bioética e Ética na Ciência do Hospital de Clínicas de Porto Alegre, Porto Alegre, Brazil

**Keywords:** telemedicine, telehealth, carbon footprint, planetary health, tertiary hospital

## Abstract

**Introduction:**

Minimizing healthcare systems’ resource footprints is crucial. To expand this focus, our objective was to assess the carbon emission reductions achievable through the introduction of telemedicine services at a prominent Brazilian tertiary hospital.

**Methods:**

This cross-section study included all patients who had remotely held appointments in a Brazilian tertiary hospital. The primary outcome was carbon emissions. The estimated carbon emissions were first calculated based on the distance between the hospital and the patient's home address. After, the calculated distance was multiplied by the amount of carbon estimated according to the type of transport used.

**Results:**

The study included 28,244 patients undergoing 52,878 remote appointments between March and December 2020, residing in 417 municipalities in Rio Grande do Sul and 80 towns in other Brazilian states. The total sum of distances and carbon gas reduction saved with the implementation of remote consultations amounted to 805,252.00 km and 939,641.94 kg of CO_2_ emissions, respectively.

**Discussion:**

Telemedicine initiatives implemented in tertiary hospitals for less than a year result in a large amount of greenhouse gas emissions saved. Telemedicine emerges as a promising strategy with significant potential to mitigate the impact on planetary health.

## 1 Introduction

Climate change is one of the foremost challenges confronting public health, particularly in urbanized areas (1, 2). As urbanization trends accelerate, projections indicate that nearly 70% of the global population will inhabit cities by 2050. This surge in density, coupled with heightened emissions, has catalyzed the emergence of many cities as focal points for hazardous air pollution (1–3).

The global vehicle population has surged across nations, contributing to escalating concerns (2–4). According to the Global Burden of Disease initiative, particulate matter pollution (PM2.5) alone was responsible for a staggering 231.9 million disability-adjusted life years (DALYs) and 7,833,220 deaths. Among risk factors, particulate matter air pollution was the leading contributor to the global disease burden in 2021, highlighting its profound impact on public health (5).

The transportation sector has significantly contributed to the escalation of carbon emissions and subsequent climate change (1). In 2021, global CO_2_ emissions from energy combustion and industrial processes saw a notable resurgence, surpassing previous records to achieve the highest annual level. The year-on-year rise of 6% compared to 2020 propelled emissions to 36.3 gigatons (6). Since CO_2_ is a significant component of global greenhouse gases, transportation carbon emissions mainly refer to CO_2_ emissions generated by transport activities. Carbon footprint research has attained tremendous popularity for improving the climate environment purposes (2, 4, 6–8).

Healthcare is one of the largest industries and considerably impacts greenhouse gas emissions, underscoring their role in exacerbating the global climate crisis (5, 7–9). To address this issue effectively, reducing patient travel has emerged as a crucial strategy. Telemedicine technology is increasingly recognized for its potential in this area. It is now considered an essential component of the new Global Health Impact Standards section within the Joint Commission International Accreditation Standards for Hospitals (10). Through telemedicine tools, substantial reductions in travel-related carbon emissions can be achieved, aligning with emission reduction objectives. The conceptual underpinnings of telemedicine have rapidly materialized into practical solutions spurred by the difficulties of the ongoing pandemic. This unforeseen paradigm shift has opened a strategic window to evaluate the advantages and hurdles of widespread telemedicine integration, offering invaluable insights to bolster community-based healthcare services (11–14). By overcoming geographical barriers, telehealth enables remote care, lowers travel expenses, decreases unnecessary emergency department visits, and early treatment and patient engagement. During the COVID-19 pandemic, it ensured care continuity, rapidly deployed medical personnel, facilitated triage, protected overloaded providers, and minimized disease transmission risks, all while maintaining patient privacy (15).

In alignment with this context, two recent systematic reviews examined the reduction of carbon footprints associated with telemedicine adoption among patients. The Rodler et al. review encompassed 48 studies, encompassing 68,465,481 telemedicine consultations, resulting in impressive savings equivalent to 691,825 tons of CO_2_ emissions and 3,318,464,047 km of avoided travel distance (16). The Van der Zee et al. included 33 articles from 1,117 records for analysis. The median round trip travel distance for each patient was 131 km [interquartile range (IQR): 60.8–351], or 25.6 kg CO_2_ (IQR: 10.6–105.6) emissions (16, 17).

Although many studies underscore the health co-benefits of reducing emissions, there still needs to be a significant gap in empirical research on large-scale, real-world tertiary hospital telemedicine applications for carbon reduction, especially in low and middle-income countries. Our study seeks to bridge this gap by offering groundbreaking insights into the environmental advantages of telemedicine, explicitly quantifying carbon emission reductions at a leading Brazilian tertiary hospital. By measuring carbon savings from remote consultations, we demonstrate telemedicine's potential as a practical and scalable solution to reduce the environmental footprint of healthcare operations. This research supports the broader goal of advancing sustainable healthcare practices, contributing to global efforts to mitigate climate change while upholding high standards of patient care.

The rationale behind this study is grounded in the urgent need to assess the environmental impact of innovative healthcare solutions, particularly considering increasing global health and environmental concerns.

## 2 Methods

### 2.1 Study design

This cross-section study estimated the carbon emission savings enabled by implementing telemedicine services leading Brazilian tertiary hospitals. This study adheres to the standards outlined in the Strengthening the Reporting of Observational Studies in Epidemiology (STROBE) guidelines, ensuring transparency, accuracy, and reliability (18).

### 2.2 Data sources and study population

In March 2020, Brazil implemented a national authorization for remote consultations in response to the escalating COVID-19 pandemic and the associated lockdown measures. This marked a pivotal shift in healthcare delivery, enabling widespread access to telehealth services nationwide.

With the COVID-19 lockdown measures, no urgent face-to-face hospital appointments were canceled at the Hospital de Clínicas de Porto Alegre (HCPA). Patients who canceled appointments due to the lockdown were contacted by phone or via the hospital app to reschedule remote consultations. To effectively conduct our study, we systematically identified eligible participants by meticulously analyzing the electronic health records (EHRs) stored within the Hospital Management Applications System Database at HCPA.

The study was conducted from March to December 2020, providing a defined timeframe to thoroughly evaluate the environmental impact of implementing remote services. This period was strategically selected to cover a complete cycle of operational practices and capture seasonal variations, ensuring the collection of robust and meaningful insights. The study included all types of visits and all specialties attended to in the hospital.

Access to the hospital's database was granted following approval from the hospital's research ethics committee.

#### 2.2.1 Inclusion criteria

All patients scheduled for telemedicine consultations during the specified period were included.

### 2.3 Setting

The research was conducted at a tertiary hospital (HCPA) in Porto Alegre, capital of Rio Grande do Sul, a southern state of Brazil. This hospital has 741 beds and provides a broad spectrum of medical services, encompassing 60 physician specialties. These services include specialized consultations for the Rio Grande do Sul state and several reference programs for patients from other states. Most of these services are delivered through the Brazilian public health system, the *Sistema Único de Saúde* (SUS).

### 2.4 Exposure

Remote consultations were extended to patients already under the hospital's care and those seeking initial consultations. These consultations adhered to the hospital's established care evidence-based protocols and were administered by a multidisciplinary team comprising doctors, physiotherapists, and nurses, tailored to each patient's specific requirements. They were carried out via a video or phone call. This service was accessible to all patients referred for treatment at the hospital. The attended consultations were included in the public funding package the HCPA receives from the Brazilian government.

### 2.5 Variables

#### 2.5.1 Carbon emission estimation

The estimated saved carbon emissions were first calculated based on the distance between the hospital, where remote consultations occurred, and the patient's home address. Different strategies were employed for this calculation, tailored to the specific location of the patient's residence.

#### 2.5.2 Distances

Patients’ addresses registered in medical records were collected to calculate the round-trip distance to the hospital. For patients from other cities, the distance was calculated from city to city, while for residents of Porto Alegre, it was measured from neighborhood to neighborhood. The total distance was determined by summing the distances from each location to HCPA, accounting for a round trip by doubling each estimated distance. Road distances were determined using data from the State's Autonomous Department of Highways (DAER) for Rio Grande do Sul patients living outside the hospital city (18). For locations outside Rio Grande do Sul and places in the same town as the hospital, distances were calculated relative to the hospital service point using the Google Maps® tool.

In the second stage of carbon emission estimation, the analysis considered the most common mode of public transportation that patients would have used if attending the consultation in person. Public transportation was the basis for all transportation estimates. This selection was based on the patient's residential location and the predominant mode of transportation typically utilized within the public health system. Carbon emissions were then estimated using standardized data from the United Kingdom Government's emissions table for 2020 (Table 1) (19). A kilogram of CO_2_ (kg CO_2_) is a basic unit commonly used for straightforward quantification. For instance, using a typical laptop yearly might produce approximately 50 kg of CO_2_ emissions. Meanwhile, a ton of CO_2_ (t CO_2_), equivalent to 1,000 kg, is often used to express more significant emissions, such as national outputs or organizational carbon footprints. To illustrate, driving an average gasoline-powered car for around 4,500 km (about 2,800 mi) would emit roughly one ton of CO_2_.

**Table 1:** Estimation of carbon emissions based on the patient's home location and the UK reference values.

Local	Carbon emission estimation
Porto Alegre	Urban bus journeys from Porto Alegre were analyzed for trips to HCPA. Carbon emissions were calculated by multiplying the distance to HCPA by 0.02872 kg of CO_2_ per kilometer (UK reference values) (19).
Municipalities in Rio Grande do Sul	For trips to HCPA from Rio Grande do Sul municipalities, a 16-seater van was considered. According to the United Kingdom table, this mode of transport (weight is between 1.74 and 3.5 tons) is considered class III vans. Carbon emissions were calculated by multiplying the distance to HCPA by 0.27171 kg of CO_2_ per km (UK reference values) (19).
Additional regions in Brazil.	
North (Acre and Rondônia), Northeast (Alagoas, Bahia, Paraíba, Sergipe, Pernambuco and Ceará), Midwest (Distrito Federal, Goiás, Mato Grosso do Sul, Mato Grosso), Southwest (São Paulo, Minas Gerais, and Rio de Janeiro).	Regions involve a long-distance plane travel, with carbon emissions computed as the distance to HCPA multiplied by 0.19085 kg of CO_2_ per Km (UK reference values) (19).
Paraná	Short-distance plane trips yield carbon emissions calculated based on the distance to HCPA multiplied by 0.2443 kg of CO_2_ per Km (UK reference values) (19).
Santa Catarina	Short-distance intercity bus trips result Carbon emissions determined by the distance to HCPA multiplied by 0.02872 kg of CO_2_ per Km (UK reference values) (19).

#### 2.5.3 Addressing biases

The selection bias was minimized by including all eligible patients.

Information bias was addressed through standardized data extraction processes. This rigorous approach ensured a comprehensive and precise selection process, allowing us to enroll patients who utilized remote appointments during the specified period.

### 2.6 Statistical methods

The hospital's data team supplied an Excel spreadsheet containing all the essential information required for this study. Using this data, we could generate estimates automatically. All data extracted from the hospital's database was included in the study, ensuring comprehensive coverage of relevant information. Using automated tools to process this data minimizes human error and ensures consistency throughout the extraction and estimation processes. Continuous variables are reported as medians and interquartile ranges (IQRs). The sample included all teleconsultations that met the eligibility criteria. Carbon emission estimates were made using Excel, Google Maps, and Statistical analyses were conducted using IBM SPSS Statistics for Windows, Version 20.0. Armonk, NY: IBM Corp.

#### 2.6.1 Patient and public involvement

This research involved the analysis of secondary data derived from existing claims data where patient or public identifiers were inaccessible. As a result, their direct involvement in the study was not feasible. However, we acknowledge and advocate for the significance of patient and public engagement in research endeavors.

## 3 Results

Between March and December 2020, 52,878 remote consultations were carried out for 28,244 patients residing in 417 municipalities within Rio Grande do Sul and 80 towns across other Brazilian states (Acre, Alagoas, Bahia, Ceará, Distrito Federal, Goiás, Minas Gerais, Mato Grosso do Sul, Mato Grosso, Paraíba, Pernambuco, Paraná, Rio de Janeiro, Rondônia, Rio Grande do Sul, Santa Catarina, Sergipe, São Paulo). Approximately 150,000 patients were seen in person in the same period at outpatient clinics.

The median distances not traveled by patients due to remote consultations from the neighborhoods of Porto Alegre to HCPA hospital, municipalities within Rio Grande do Sul to Porto Alegre, and from the municipalities of other states to Porto Alegre were 20.00 km (IQR25-75 12.00–28.00), 515.00 km (IQR25-75:250.50–783.00), and 1,320.00 km (IQR25-75: to 878.00–4,952.00), respectively. The total number of distances saved with remote consultations amounted to 805,252 km.

Regarding carbon gas reduction from the neighborhoods of Porto Alegre to HCPA hospital, from municipalities within Rio Grande do Sul to Porto Alegre, and from the capitals of other states to Porto Alegre were 51.26 kg of CO_2_ (IQR25-75: 7.36–146.42), 876.26 kg of CO_2_ (IQR25-75: 348.33–2,455.58), and 13,286.21(IQR25-75: 3,868.69–28,044.93), kilograms of CO_2_ respectively. The total sum of carbon gas reduction saved with the implementation of remote consultations amounted to 939,641.94 kg of CO_2_.

## 4 Discussion

Our study reveals that over 50,000 remote consultations in a Brazilian tertiary hospital significantly reduced 939,641.94 kg of CO_2_ emissions. These findings position telemedicine as an effective strategy for sustainable healthcare, highlighting its role at the intersection of healthcare innovation and environmental sustainability. It highlights telemedicine's role in regions with high emissions and scarce resources, advocating its integration into standard healthcare protocols. The evidence provided can help policymakers and healthcare leaders expand telemedicine use, aligning environmental health goals with quality and accessible care. By filling a critical research gap with empirical data from a low- to middle-income country, this study underscores telemedicine's importance in improving healthcare efficiency and environmental sustainability in the fight against climate change.

The environmental impact of telemedicine aligns with the United Nations 2030 Agenda, especially at the intersection between health and sustainability, playing a significant role in promoting the Sustainable Development Goals (SDGs) (20). By reducing the need for physical travel for consultations, telemedicine directly contributes to reducing greenhouse gas emissions and the more efficient use of resources such as energy and infrastructure, supporting SDG 13 (Climate Action). Moreover, expanding access to healthcare, especially for populations in remote or underserved areas, addresses SDG 3 (Good Health and Well-being), promoting equity in access to quality medical services. This approach reinforces SDG 9 (Industry, Innovation, and Infrastructure) by integrating advanced technological solutions into health systems, creating a more resilient and accessible network. Thus, telemedicine improves public health and drives a sustainable development model that balances social, economic, and environmental needs.

Beyond the purely SDG, our results should be discussed in light of social theories with an interdisciplinary approach that can broaden our understanding of the benefits of implementing remote consultations. They offer lenses through which the research problem can be examined and interpreted, ensuring a systematic and analytical approach to understanding complex phenomena.

Our results suggested that telemedicine is an effective strategy for a more sustainable healthcare system by reducing carbon emissions, besides previous evidence lowering costs and improving access, aligning with the Triple Bottom Line and Ecological Modernization Theory. However, successful implementation requires careful change management, addressing emotional resistance, and adapting workflows, as framed by models like Kübler-Ross's Change Curve, Lewin's model, and Normalization Process Theory. Stakeholder Theory further emphasizes the importance of balancing the needs of patients, employees, and the environment, necessitating a holistic approach that integrates technical infrastructure, social adaptation, and strategic planning to achieve genuinely sustainable healthcare practices, as demonstrated by HCPA's experience (21–28).

HCPA's strategic planning, considering various operational factors, estimated that 20% of consultations could be conducted remotely each year. Extrapolating the findings of this study suggests a potential reduction in greenhouse gas emissions of more than 2,131,420 per year of CO_2_. HCPA is a highly complex hospital and a national reference in multiple specialties, receiving patients from various Brazilian states. Therefore, these results may be generalizable to other countries that refer patients to specialized centers.

While the study's pragmatic approach enables a practical assessment of carbon emission reductions, it is essential to acknowledge that potential inaccuracies in data address and assumptions about transportation modes could influence distance and emission calculations. Without specific and data on individual patient travel modes from records, there is inherent uncertainty in the accuracy of the carbon emission reduction calculations, which may impact the reliability of the study's conclusions. Given the importance of the data, we recommend that future research incorporate questionnaires to gather this information, considering individual patient travel data and additional environmental factors.

While sensitivity analyses or validation procedures were not conducted in this study, the findings were based on comprehensive data extraction and rigorous analysis methods. Future research could benefit from further incorporating these techniques to ensure the results’ robustness and generalizability. However, to mitigate this bias, we employed conservative estimates by assuming public transportation in all cases, even though some patients might have used private transportation. On the other hand, given that these estimates are based solely on public transit, the results should be extrapolated with caution. Assuming public transportation as the default mode could add depth to the analysis. Nonetheless, most patients at HCPA rely on this mode of transport. A further limitation of our study is the reliance on carbon emission reference data from the United Kingdom Government, as Brazil lacks regularly updated data covering a comprehensive range of vehicle options. However, the UK data encompasses various vehicles and brands, including those commonly used in Brazil.

Another limitation inherent to national ecological studies employing various modes of transportation is the utilization of diverse methodologies for estimating CO_2_ emissions. The lack of internet access and familiarity with telehealth services could pose a significant limitation to the generalizability of our results. However, according to the 2023 National Household Sample Survey on technology, 93% of the Brazilian population now has internet access.

A scoping review highlighted the current gaps and limitations in environmental assessment frameworks for evaluating the carbon footprint of digital health interventions in healthcare. Despite screening many studies, only a tiny fraction of 13 articles provided relevant data for analysis. Among the studies reviewed, there was a notable absence of standardized methods or validated tools for systematically assessing the environmental sustainability of digital health interventions throughout their lifecycle. Furthermore, no validated systems-based approach was identified for evaluating and validating digital health interventions. Interestingly, the review found that only three studies focused on digital health interventions within hospital settings, and all these studies were conducted in high-income countries. This suggests a significant gap in research regarding the environmental impact of digital health interventions, particularly within healthcare facilities in middle-income settings (29, 30).

The significance of this study extends beyond academic interest; it has practical implications for healthcare providers, policymakers, and hospital administrators. By demonstrating measurable environmental benefits, the research supports the case for expanding telemedicine services, promoting policy changes, and encouraging sustainable healthcare practices. Additionally, the findings contribute to theoretical frameworks by integrating environmental perspectives into telemedicine studies, enriching the discourse on sustainable healthcare innovations. Ultimately, this study advances academic understanding and offers tangible benefits for enhancing healthcare delivery and achieving environmental goals.

The study strongly supports the strategic adoption of telemedicine as a transformative approach. It underscores the necessity of innovative approaches like telemedicine in the global effort to combat climate change, emphasizing healthcare efficiency and environmental sustainability benefits. To maximize these benefits, we recommend that healthcare providers integrate telemedicine into routine care and utilize data analytics to tailor services for better patient outcomes. Policymakers should develop supportive regulations and invest in digital infrastructure to ensure equitable access while emphasizing telemedicine's environmental benefits to incentivize adoption. Hospital administrators can implement scalable telemedicine platforms and foster multidisciplinary teams for comprehensive care, complemented by systems to monitor impact. Telemedicine offers patients enhanced access to services, convenience, reduced travel, and improved health outcomes through more frequent monitoring and follow-up care. By aligning efforts across these groups, telemedicine can be effectively scaled, benefiting healthcare systems and the environment.

Although telehealth offers benefits such as reduced travel, it also introduces new sources of energy consumption related to data storage, transmission, and device usage. Addressing these energy demands through technological and operational improvements is vital for achieving sustainable telehealth solutions and equitable access (13). Specific medical procedures or interventions, such as surgical procedures or hands-on treatments, cannot be performed remotely, limiting the scope of conditions that can be effectively managed through telehealth.

### 4.1 Action research

An action research approach could also innovate telemedicine practices to further reduce healthcare's carbon footprint. This method fosters collaboration between researchers and practitioners to address issues, implement solutions, and refine strategies. For example, hospitals might work with transportation planners and environmental scientists to minimize travel-related emissions or optimize telemedicine platforms to decrease the necessity for follow-up in-person visits. Action research's participatory nature ensures practical relevance and drives systemic change, making it well-suited for tackling complex challenges like planetary health.

## Data Availability

The raw data supporting the conclusions of this article will be made available by the authors, without undue reservation.
